# Novel mimetic tissue standards for precise quantitative mass spectrometry imaging of drug and neurotransmitter concentrations in rat brain tissues

**DOI:** 10.1007/s00216-024-05477-5

**Published:** 2024-08-10

**Authors:** Kenichi Watanabe, Sayo Takayama, Toichiro Yamada, Masayo Hashimoto, Jun Tadano, Tetsuya Nakagawa, Takao Watanabe, Eiichiro Fukusaki, Izuru Miyawaki, Shuichi Shimma

**Affiliations:** 1 Preclinical Research Unit, Sumitomo Pharma Co., Ltd., Osaka, Japan; 2 Research Planning & Coordination, Sumitomo Pharma Co., Ltd., Osaka, Japan; 3 Drug Research Division, Sumitomo Pharma Co., Ltd., Osaka, Japan; 4 Department of Biotechnology, Graduate School of Engineering, Osaka University, Osaka, 565-0871 Japan; 5 Institute for Open and Transdisciplinary Research Initiatives, Osaka University, Osaka, Japan; 6 Osaka University Shimadzu Omics Innovation Research Laboratory, Osaka University, Osaka, Japan

**Keywords:** Quantitative mass spectrometry imaging, Matrix-assisted laser desorption/ionization, Clozapine, Neurotransmitters, Brain

## Abstract

Understanding the relationship between the concentration of a drug and its therapeutic efficacy or side effects is crucial in drug development, especially to understand therapeutic efficacy in central nervous system drug, quantifying drug-induced site-specific changes in the levels of endogenous metabolites, such as neurotransmitters. In recent times, evaluation of quantitative distribution of drugs and endogenous metabolites using matrix-assisted laser desorption/ionization (MALDI)-mass spectrometry imaging (MSI) has attracted much attention in drug discovery research. However, MALDI-MSI quantification (quantitative mass spectrometry imaging, QMSI) is an emerging technique, and needs to be further developed for practicable and convenient use in drug discovery research. In this study, we developed a reliable QMSI method for quantification of clozapine (antipsychotic drug) and dopamine and its metabolites in the rat brain using MALDI-MSI. An improved mimetic tissue model using powdered frozen tissue for QMSI was established as an alternative method, enabling the accurate quantification of clozapine levels in the rat brain. Furthermore, we used the improved method to evaluate drug-induced fluctuations in the concentrations of dopamine and its metabolites. This method can quantitatively evaluate drug localization in the brain and drug-induced changes in the concentration of endogenous metabolites, demonstrating the usefulness of QMSI.

**Graphical Abstract:**

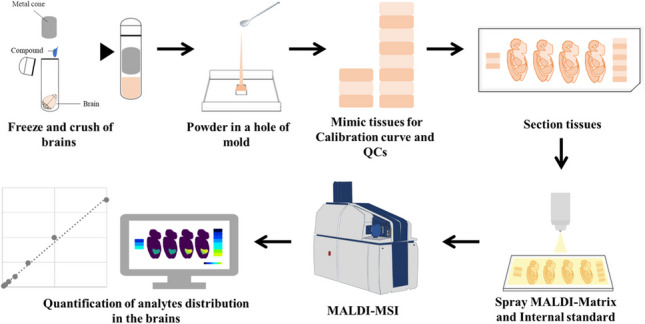

**Supplementary Information:**

The online version contains supplementary material available at 10.1007/s00216-024-05477-5.

## Introduction

Mass spectrometry imaging (MSI) is a powerful tool that facilitates drug discovery research by enabling direct acquisition of spatial information on various molecules, especially in pharmacokinetic and toxicological studies, which is not possible using conventional liquid chromatography-mass spectrometry (LC/MS) [1–4]. Matrix-assisted laser desorption/ionization (MALDI)-mass spectrometry is the most commonly used method for the determination of peptides [5, 6], lipids [7, 8], bile acids [9], glycolytic molecules [10], glycans [11], proteins [12], and drugs and their metabolites [2–4]. Methods using derivatization reagents have recently been developed for assessing the distribution of trace molecules such as neurotransmitters in the brain [13–15] and are finding application in studies aimed at understanding molecular mechanisms in the brain [16–19]. A thorough comprehension of complex mechanisms underlying drug action in the brain is crucial in central nervous system (CNS) drug discovery research. In this regard, MSI is promising for investigating the spatial variation of neurotransmitters induced by drugs.

Although MSI has been developed as a research tool for drug discovery, quantitative MSI (QMSI) is an emerging field with regard to technical and regulatory aspects [20–22]. This is because in MALDI-MSI, foreign molecules in tissue samples are simultaneously ionized with in the MALDI source, leading to ionization suppression becoming a technical challenging, and the heterogeneous distribution of biomolecules in tissue adds complexity to the quantification of target molecules [23].

In QMSI, three different models for a quantitative measurement of drug are typically used for the calibration, namely the tissue extinction coefficient (TEC) model [24], the dilution series model [25–27], and the mimetic tissue model [28–30]. In the TEC model, the concentration of drug in a tissue sample is determined by spraying a control tissue section with a known concentration of drug solution and calculating the local correction factor (drug intensity on the tissue divided by the drug intensity on the glass slide). Although preparation of samples in this method is simple, the difference in extraction efficiency of compounds from different tissue regions is not taken into consideration. The dilution series model uses a calibration curve generated from known concentrations of drug solution spotted on a tissue section of interest, and it is used for the quantitation of neurotransmitters as well as drugs. While ionization effects on the tissue can be considered in this model, the degree to which the extraction efficiency of each locally placed drug solution within the actual tissue can be mimetic remains unclear. In the mimetic tissue model, an “in the tissue” environment is simulated. This model uses blocks of tissue homogenates to which different concentrations of a drug frozen into a mold have been added to quantify the concentrations of the drug in tissue sections. The use of drug-spiked homogenates frozen using the mold allows mimetic the dosed tissue environment and has been shown to be able to correct for tissue-specific matrix effects and extraction efficiency [31].

The mimetic tissue model has been reported to be quantitatively more reliable than the dilution series model [32]. However, the mimetic tissue model requires substantial amounts of blank tissue, a multistep process, and considerable effort to prepare tissue homogenates blocks [32]. Recently, Lamont et al. reported a simple method for creating mimetic samples using molds constructed with a 3D printer [23], and Nagano et al. proposed a method to improve handling and creation efficiency of mimetic tissues [33, 34]. Although further efforts are needed to develop the technical and regulatory aspects of QMSI, these calibration methods are steadily advancing the field of QMSI research.

In this study, we developed an improved mimetic tissue model by partially modifying the mimetic tissue method established by Nagano et al. [34]. and verified the accuracy of the calibration curves and quality control samples (QCs) using clozapine (CLZ) as a compound for verification. We quantified CLZ concentrations in the brain of CLZ-treated rats with QMSI and compared them with the concentrations determined using LC/MS. Furthermore, we applied this mimetic tissue model to the quantification of neurotransmitters in the rat brain using a derivatization reaction to quantify the concentration of dopamine (DA) and its metabolites in the brain, and to evaluate the fluctuations in their concentrations caused by the drug. In CNS drug discovery research, it is useful to measure changes in DA, 3-MT, and HVA concentrations, which serve as biomarker of DA metabolism. This allows for the assessment of drug-induced activation of DA neurons or the progression of diseases affecting DA neurons [35]. Based on these studies, we demonstrated that QMSI holds the potential to ensure accurate quantification of MSI results, addressing a significant challenge in the localization of drugs of the brain and spatial metabolomics.

## Experimental section

### Chemicals

2,5-Dihydroxybenzoic acid (DHB) was purchased from Sigma-Aldrich (St. Louis, MO, USA). 4-(Anthracen-9-yl)-2-fluoro-1-methylpyridin-1-ium iodide (FMP-10) was purchased from Tag-ON (Uppsala, Sweden). Clozapine and DA were purchased from Tokyo Chemical Industry Co., Ltd. (Tokyo, Japan). Homovanillic acid (HVA) was purchased from Nacalai Tesque, Inc. (Kyoto, Japan). 3-Methoxytyramine (3-MT), CLZ-d4, DA-d4, 3-MT-d4, and HVA-d5 were purchased from Cambridge Isotope Laboratories Inc. (Cambridge, MA, USA). Water, methanol, and ethanol were obtained from Kanto Chemical Industry Co, Ltd. (Tokyo, Japan). Mayer’s hematoxylin solution was obtained from FUJIFILM Wako Chemicals (Tokyo, Japan).

### Animal experiments

Eight-week-old male Sprague–Dawley (SD) rats (*n* = 3 per group) were maintained on a 12-h light/dark cycle (08:00/20:00 h) at 22 °C with free access to food and water. For CLZ measurements, rats received a single intraperitoneal dose of vehicle (*n* = 1) or 60 mg/kg of CLZ (*n* = 3). For neurotransmitter measurements, rats received a single intraperitoneal dose of vehicle (*n* = 2) or 40 mg/kg of CLZ (*n* = 2). Untreated 8-week-old male SD rats were used to prepare mimetic samples. The rats were quickly decapitated after euthanasia, and their brains were promptly removed (within 60 s), stabilized with Stabilizer T1™ (Denator AB, Uppsala, Sweden), and frozen in liquid nitrogen. Brain or mimetic tissue was sectioned at − 20 °C into 10-μm slices using a Leica CM1950 cryostat (Leica Biosystems, Nussloch, Germany). The sections were mounted on MAS Coat Sliding Glass™ (Matsunami Glass Industries, Osaka, Japan) or PEN membrane-coated glass slides (Leica Microsystems K.K., Tokyo, Japan) and stored at − 80 °C prior to use. Before use, the sections were slowly brought to room temperature (20–25 °C) in a desiccator. The Animal Care Committee of Sumitomo Pharma Co., Ltd. approved all animal procedures described in this study (AN13785-B00).

### Preparation of CLZ-containing mimetic tissue block from powdered tissues

**Fig. 1:**
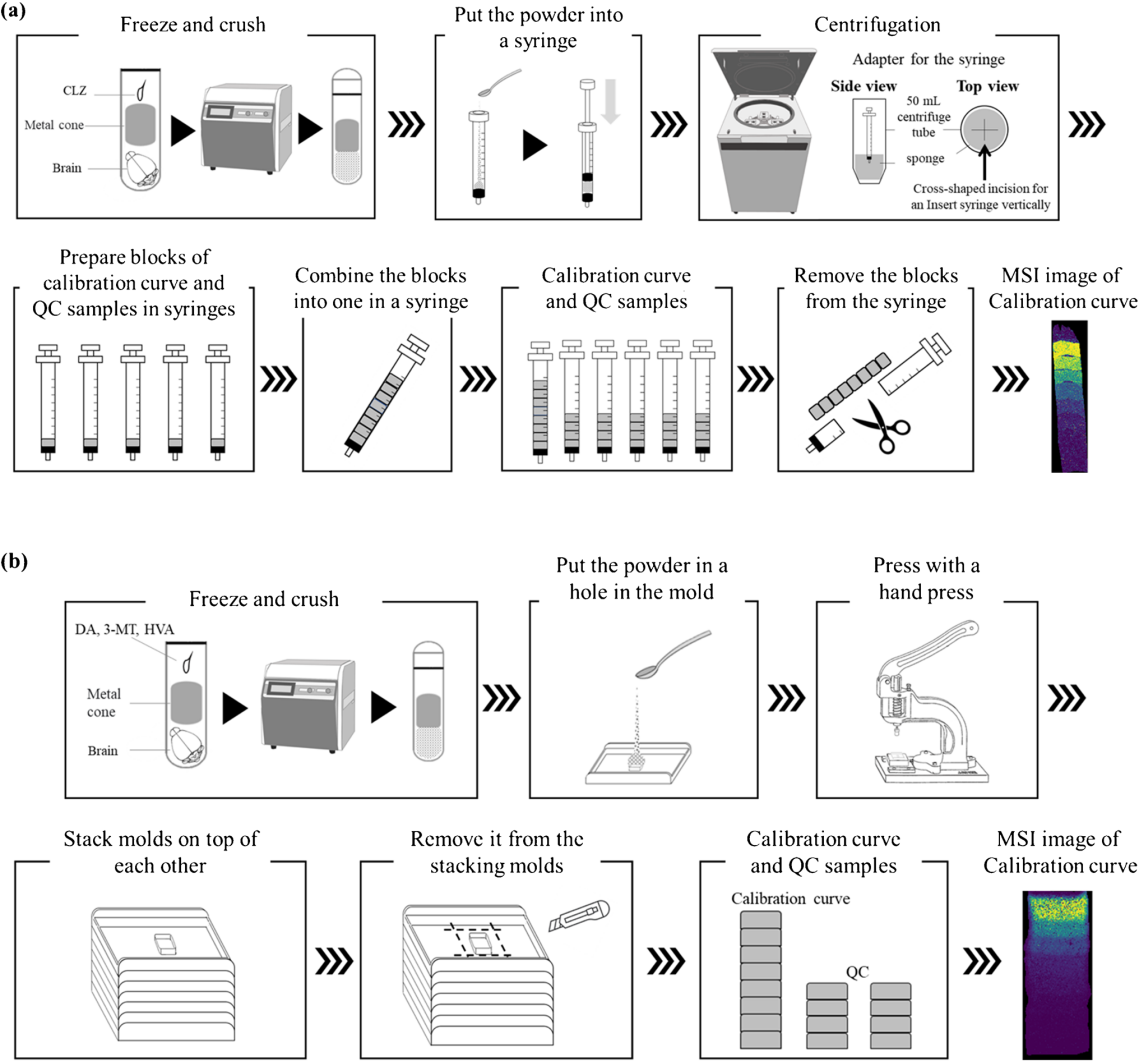
The workflow for construction of a mimetic model. **a** Mimetic tissue models construction step for Clozapine (CLZ) using syringes. **b** Mimetic tissue models construction step for dopamine (DA), 3-methoxytyramine (3-MT), and homovanillic acid (HVA) using molds

The steps in the preparation of the model are schematically shown in Fig. 1a. The brain and CLZ in methanol standard solutions were added to a multi-bead shocker tube (Yasui Kikai Corporation, Osaka, Japan) to obtain concentrations of 0, 1, 2, 5, 10, 20, 40, 60, and 80 μg/g. Ten microliter or less of each prepared standard solution was added per gram of the brain tissue, and the volume of standard solution added was 1% (v/w) or less of the weight of the brain tissue. Each multi-bead shocker tube placed in sample holders was cooled in liquid nitrogen until use. The samples in dedicated tubes were placed in a multi-bead shocker (Yasui Kikai Corporation, Osaka, Japan) and crushed twice (725 g × 20 s). Each sample holder was then recooled in liquid nitrogen and the sample was crushed again twice under the same conditions to obtain brain tissue powder prepared at different concentrations. The powder was quickly added into a syringe (1 mL, Terumo^®^ Syringe) with the seal removed from the plunger, slightly melted at room temperature, and centrifuged (2900 g × 5 min, 4 °C, High-Speed Micro Centrifuge Model CF16RN, Eppendorf Himac Technologies Co. Ltd.) with an adapter designed for the syringe. After centrifugation, the slightly melted powder was refrozen on dry ice. The same procedure was performed for each calibration standards and QCs of the 28 blocks. The series of blocks with different concentrations, ranging from low to high, were stacked in a syringe and then centrifuged (2900 g × 5 min, 4 °C) with an adapter designed for the syringe. All calibration standards and QC samples (5, 20, and 60 μg/g) were placed in a syringe; the entire syringe was cooled in a − 80 °C freezer. After sufficient cooling, the syringe tip was cut with a dog nail clipper, and a cylindrical brain mimetic tissue sample was quickly ejected with a new plunger on dry ice.

### Preparation of DA, 3-MT, and HVA-containing mimetic tissue block

A schematic of this procedure is shown in Fig. 1b. Mixed standard solutions of DA, 3-MT, and HVA were added to the multi-bead shocker tube, in proportion to the weight of the rat brain, with only the cerebellar portion being used. The calibration curve concentrations of DA and HVA were 0, 1.56, 3.13, 6.25, 12.5, 25, 50, and 100 μg/g, and the concentration of 3-MT was half that of these concentrations. The QC sample concentrations of DA and HVA were 0, 3.13, 12.5, and 50 μg/g, and the concentration of 3-MT was half of these concentrations. Similarly, mixed standard solutions were prepared and added to 1 g of the brain tissue. In this case, 10 µL of each prepared standard solution was added to 1 g of the brain tissue, and the volume of the added standard solution was kept below 1% (v/w) of the brain tissue weight. Each multi-bead shocker tube placed in sample holders was cooled in liquid nitrogen until use. The samples in dedicated tubes were placed in the multi-bead shocker and crushed twice (725 g × 20 s). Thereafter, each sample holder was recooled in liquid nitrogen and crushed twice under the same conditions. The base mold (M475-1, AS ONE Corporation, Osaka, Japan) and adapter designed for the hand press were cooled with dry ice; the base mold was filled with the brain tissue powder, covered with a new base mold, and compressed with a hand press. A base mold, with the bottom cut out, was placed on top of the base mold on dry ice, the brain tissue powder of different concentrations was placed on top, and the same operation was performed. Calibration standards of all the brain tissue powder were stacked and allowed to stand at 4 °C for 30 min, and then each base mold was thoroughly cooled in a − 80 °C freezer. The QCs were stacked one by one in the same way as in the case of calibration standards, with three concentrations in one block, and then allowed to stand at 4 °C for 30 min, and each base mold was cooled in a − 80 °C freezer. After sufficient cooling, the base molds were removed individually to obtain square-column-shaped mimetic brain tissue samples.

### MALDI-MSI

For CLZ analysis, DHB (30 mg/mL, acetonitrile/water/trifluoroacetic acid, 70/30/0.2; v/v/v) was uniformly coated onto the tissue sections using the M5 TM-Sprayer™ (HTX Technologies, Chapel Hill, NC, USA) under the following conditions: nozzle temperature was set at 65 °C, the reagents were sprayed pneumatically (10 psi of N_2_) in 24 passes at a linear velocity of 120 cm min^−1^ with 2-mm track spacing and a flow rate of 50 μL min^−1^. The amount of matrix per unit area of the coated matrix was 10 μg/mm^2^. The internal standard solutions (1 μg/mL CLZ-d4 solution in 80% methanol) were sprayed over the tissue sections using the same condition as described above before DHB use. For DA and DA metabolite analysis, FMP-10 (2.2 mg/mL, 70% acetonitrile) was uniformly coated onto the tissue sections using M5 TM-Sprayers under the following conditions [13]: nozzle temperature was set at 80 °C, the reagents were sprayed pneumatically (6 psi of N_2_) in 30 passes at a linear velocity of 110 cm min^−1^ with 2-mm track spacing and a flow rate of 70 μL min^−1^. The amount of matrix per unit area of the coated matrix was 1.68 μg/mm^2^. The internal standard solutions (1 μg/mL DA-d4, 1 μg/mL 3-MT-d4, and 10 μg/mL HVA-d5 solution in 80% methanol) were sprayed over the tissue sections using the same condition as described above before FMP-10 derivatization.

MALDI-MSI measurements were performed using a 7 T scimaX MRMS Fourier transform ion cyclotron resonance (FT-ICR) mass spectrometer equipped with a Smartbeam II 2 kHz laser (Bruker Daltonics GmbH, Bremen, Germany) in the positive ion mode. The laser power was optimized at the start of each experiment and maintained at a constant level throughout the MALDI-MSI experiments. The measurements were carried over an m/z range of 300–750. The mass resolution was set to a high mass resolution mode (200,000 FWHM at *m/z* 400) and the spatial resolution was 70 μm. All methods used were calibrated externally using sodium formate. In addition, ESI-L Low Concentration Tuning Mix (Agilent Technologies, Santa Clara, CA, USA) with flow injection for locked mass calibration was used to adjust the mass calibration scale. Tissue sections were analyzed randomly to prevent possible biases, such as matrix degradation and variations in MS sensitivity. Each calibration curve, QC, and rat brain sagittal section required approximately 4–12 h (in the case of DHB) to 3–9 h (in the case of FMP-10) of the measurement time depending on the sample size. MALDI-MSI data were visualized using the SCiLS lab Pro™ software program (version 2021b; Bruker Daltonics GmbH, Bremen, Germany). The *m/z* values for CLZ, DA, HVA, 3-MT, and their corresponding stable isotopes were determined based on the chemical formula and previous reports [13, 30].

### Calculation of concentration

The IMAGEREVEAL MS software (Shimadzu, Kyoto, Japan) was used to select regions of interest (ROIs) in the acquired MS images. The matrix effect was corrected by using uniformly distributed isotope-labeled analogs of the target analytes. A calibration curve was generated by peak ratios (analyte/stable isotope), which were calculated from the calibration samples. A blank sample and seven calibration standard samples were measured, and a weighted linear regression analysis was performed to confirm linearity. The calibration curves were used to calculate the molecular concentration of the target site and the QC concentrations. If the concentration of the target molecule at the target site did not fall within the linear range of the set concentration, it was calculated by extrapolation.

### Evaluation of the quantitativeness of CLZ-containing mimetic tissue

Six mimetic tissue sections were placed on MAS Coat Sliding Glass™ (Matsunami Glass Industries, Osaka, Japan) for calibration (*n* = 1) and QC (*n* = 5) (Figure S1a). We focused on the technical evaluation of QMSI and for technical verification of QMSI, we evaluated the dynamic range, linearity (*R*^2^), limit of detection (LOD), relative error (%RE), relative standard deviation (%RSD), and stability at − 80 °C. The %RE and %RSD were calculated for the concentration of the molecule of interest in each concentration layer of the mimetic tissue sections. The %RE was calculated as follows: %RE = ([measured value] − [theoretical value]) / [theoretical value] × 100. The %RSD was calculated as follows: [standard deviation] / [mean concentration] × 100%.

### Quantification of CLZ and neurotransmitters in rat brain sections

For CLZ quantification, frozen sections of mimetic tissue blocks for CLZ-containing calibrations and QC samples mimetic tissue blocks were prepared for rat brains treated with CLZ or vehicle (CLZ, *n* = 3; vehicle, *n* = 1) and were placed on MAS Coat Sliding Glass™ (Matsunami Glass Industries, Osaka, Japan) (Figure S1b). For neurotransmitter quantification, frozen sections of DA, 3-MT, and HVA-containing calibrations, and QC samples of mimetic tissue blocks were prepared from the rat brain treated with CLZ or vehicle (CLZ, *n* = 2; vehicle, *n* = 2) and placed on MAS Coat Sliding Glass™ (Figure S1c). These samples were measured on the same glass slides in the order of calibration curve, sample, and QC sections, and the samples were sandwiched between samples of known concentrations to confirm the stability of the operation during measurement using the calculated calibration curves and QC accuracy. The concentration of the target molecule in the tissue sections was determined using the calibration curve.

### Statistical analyses

The significance of accumulation changes of CLZ between different brain region groups (comparing cortex with cerebellum, and thalamus with cerebellum) was analyzed using an unpaired *t*-test in Microsoft^®^ Excel2019. The *p*-values less than 0.05 are highlighted in Table 4.

### Laser capture microdissection (LCM)-LC/MS

Calibrations and QCs prepared with mimetic tissue, and each region of the rat brain (cortex, thalamus, and cerebellum) were collected in PCR tubes (Thermo Fisher Scientific, CA, USA) using LMD7 (Leica Microsystems GmbH, Wetzlar, Germany) (Figure S3). The collected tissue was incubated and extracted in CLZ-d4-containing methanol (100 μL) with sonication. The tubes were centrifuged (20,900 g for 5 min at 4 °C). The supernatant was analyzed using an LC/MS system consisting of a Nexera™ (Shimadzu, Kyoto, Japan) and a 5500QTrap (ABSCIEX, Toronto, Canada). Separation was performed on a Cadenza C18 column (3 μm, 2.1 mm I.D. × 50 mm, Imtakt Corp., Kyoto, Japan) at 40 °C and a flow rate of 400 μL/min with 0.1% formic acid water as mobile phase A and acetonitrile as mobile phase B. The gradient conditions were changed from 90/10 to 10/90 in 3 min for the A/B ratio, held at 10/90 from 3.0 to 3.5 min, immediately returned to 90/10, and held for 1.5 min. The overall runtime was 5 min and MRM measurements were performed in the positive ion mode (MRM transitions, CLZ *m/z* 327 → 270, CLZ-d4 *m/z* 331 → 270). Data acquisition and interpretation were performed using the Analyst^®^ 1.7 Software (AB SCIEX, Concord, Canada) and Microsoft^®^ Excel2019. The assessed concentration was corrected by each area value obtained using LMD7 (Figure S3).

## Results and discussion

### Advantage of the new mimetic tissue model for QMSI

In this study, it is crucial to use powdered tissue as the method. Additionally, to confirm the scalability of the technique, two different filling methods were considered. While both methods are nearly equivalent in terms of creating calibration curves, the syringe method requires centrifugal compaction, which can be time-consuming and occasionally results in fragile blocks when compacting powder. This fragility can make subsequent sectioning challenging, potentially due to insufficient compaction during centrifugation. On the other hand, the mold method has improved compaction during the compaction process, allowing for convenient creation by layering the powder while using a tablet press. According to conventional mimetic tissue models, it is recommended to prepare mimetic samples using tissue homogenization with a water-soluble solvent concentration of less than 3% in order to obtain an ideal mimetic tissue [30]. However, it is very difficult to avoid the presence of bubbles due to the high viscosity of the tissue homogenate. These bubbles act as voids within the mimetic tissue model and can alter the matrix effect, potentially rendering them unusable for calibration curves or QCs. In contrast, in our method, because compounds can be mixed uniformly in the tissue as a powder, it is easy to build the homogeneous block and no voids are created due to the presence of air bubbles (Fig. 1a and b). Furthermore, our method allows the use of organic solvents for compound addition because the amount of solvent used is kept below 1% (w/v) of the brain tissue weight, and the brain tissue is used as a powder. This is especially advantageous when examining candidate drug compounds in the CNS field, which contains many lipophilic compounds. The blocks of each set concentration can be easily stacked, and calibration curves or QC can be obtained in a single sectioning. The thickness of each concentration layer can be easily controlled, which saves space for the calibration curve and QC samples to be placed on a single glass slide, allowing more samples to be placed.

### Selectivity, linearity, and accuracy using calibration curve samples

**Fig. 2:**
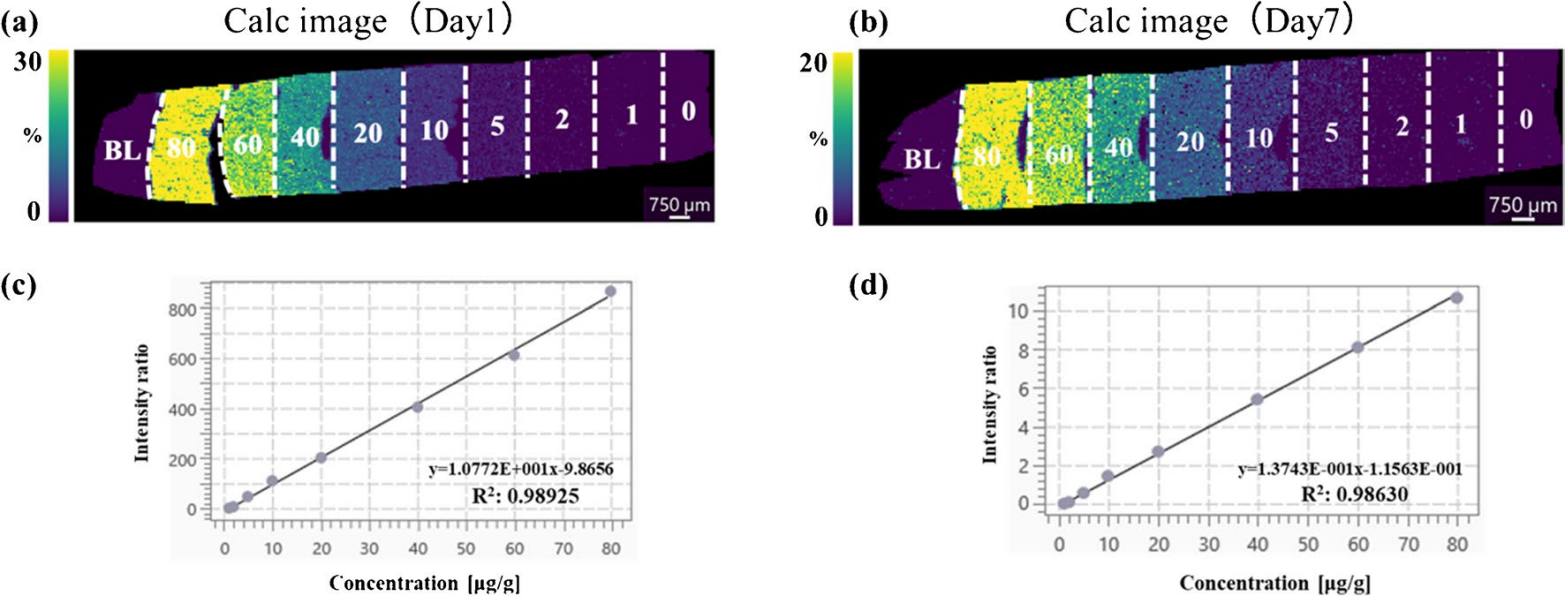
Calibration curves (Calc) on day 1 and day 7 of CLZ with mimetic tissue models. **a** Calculated image of day 1. **b** Calculated image of day 7. **c** Calibration curves of CLZ on day 1. **d** Calibration curves of CLZ on day 7. Images of CLZ (*m*/*z* 327.136, scaled from 0 to 30%) were acquired with a spatial resolution of 70 μm and normalized to the signal of the isotope-labeled standard (CLZ-d4, *m*/*z* 331.165). The region by white dashed lines indicates the respective concentration layer used for constructing the calc. weighted least squares calibration curves (solid lines) was applied to correlate the average ion intensity of each layer of the mimic model to its corresponding setting concentration

No diffusion of CLZ from the highest concentration in the mimetic tissue model to the blank tissue was observed, and no cross-contamination between the mimetic samples of each concentration was observed in the calibration curve samples (Fig. 2a and b). The ion intensity of the 0 μg/g samples in the blanks and calibration curves was approximately 1/14–1/8 of the lowest concentration (the lower limit of quantitation, LLOQ). Good linearity (*R*^2^ > 0.98) and accuracy were observed for the calibration curves prepared from samples prepared on days 1 and 7 after sectioning (Fig. 2c, d; Table 1). The approach of spraying a stable isotope standard solution is known to improve the quantitation in MSI [29]. The effect of this method was also examined beforehand using CLZ-d4, and it was confirmed that the accuracy of quantitation was improved (Figure S2). On days 1 and 7, comparable linearity was observed; however, there was a substantial difference in the ratio, and the sensitivities of CLZ and CLZ-d4 were significantly different (Fig. 2c and d). Thus, it is considered important to establish appropriate calibration curves that can correct for the “inter-day variation” that may occur in MALDI-MSI when performing measurements across different days and comparing the results. The QMSI methodology using calibration curves meets these requirements and is considered to be useful.

**Table 1:** Calibration curve characteristics of clozapine (CLZ) measured using MALDI-MSI

CLZ	Day1		Day7	
Equation	y = 1.0772E + 001x-9.8656		y = 1.3743E-001x-1.1563E-001	
*R*^2^	0.989		0.986	
Sample type	Concentration (μg/g)	%RE	Concentration (μg/g)	%RE
Calibration curve	1.08	7.92	1.11	10.9
	1.63	− 18.6	1.57	− 21.5
	5.15	2.92	4.71	− 5.72
	11.0	10.2	11.0	9.67
	19.8	− 1.13	20.3	1.58
	38.3	− 4.17	40.2	0.515
	57.5	− 4.21	59.9	− 0.238
	81.2	1.48	78.1	− 2.32

CLZ lower limit of quantification (LLOQ) was 1 μg/g. It was selected with an analyte response which is at least 5 times the blank response

### Accuracy, precision, and stability using QC samples

**Fig. 3:**
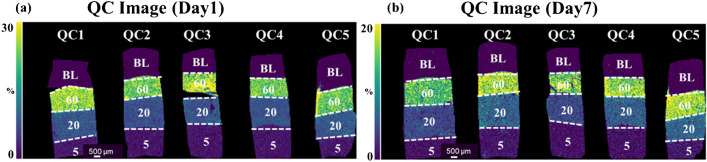
Images of quality control (QC) samples on days 1 and 7 of clozapine (CLZ) treatment of mimetic tissue models. QC image on **a** day 1 and **b** day 7. Images of CLZ (*m*/*z* 327.136, scaled from 0 to 30%) were acquired with a spatial resolution of 70 μm and normalized to the signal of the isotope-labeled standard (CLZ-d4, *m*/*z* 331.165). The region bordered by white dashed lines indicates the respective concentration layer used for constructing the QC samples

To confirm the reliability of the measurements using this method, five QC samples of three concentrations were prepared in one batch of measurements, and MSI measurements were performed with the calibration curve samples to calculate %RE and %RSD based on *n* = 5 replicates (Fig. 3a, b; Table 2). Both days 1 and 7 after sectioning, the %RE and %RSD were less than ± 20%. There was a slight increase in %RSD on day 7 and it cannot be denied that impurities affecting ionization during MS measurements of CLZ and CLZ-d4 may have changed between days 1 and 7. The %RE and %RSD values obtained in this measurement were comparable to the acceptance criteria for ICH M10 guideline [36]. This guideline provides recommendations for accurately measuring drug concentrations in biological samples used in drug development. In addition, since MSI measurements require fewer separation steps than LC/MS, past report indicated that a variation of 13 to 27% RSD was considered acceptable for MSI measurement [29]. In light of this, it is possible to consider that the current results for measuring CLZ-containing brain mimetics tissue fall within the acceptable range of variability of measurements observed in MSI (Table 2). These results indicated that the samples can remain stable at − 80 °C for 1 week after sectioning, when evaluating the distribution differences of CLZ in the brain. Although there are currently no guidelines for quantitative measurements in QMSI, the application of the ICH M10 guideline [36] to QMSI is controversial. Due to the fact that QMSI methods generally exhibit more variability compared to typical LC/MS methods [29], there is a need for further knowledge accumulation regarding QMSI with various compounds. Currently, it is essential to determine the appropriate level of precision based on the objectives of quantitative analysis in each study.

**Table 2:** Quality control characteristics of clozapine (CLZ) measured using MALDI-MSI

CLZ conc. (μg/g)	Day1						Day7					
	%RE					%RSD	%RE					%RSD
	QC1	QC2	QC3	QC4	QC5		QC1	QC2	QC3	QC4	QC5	
5(LQC)	13.8	− 4.78	− 0.284	3.05	12.7	7.75	− 17.2	8.15	− 17.5	− 8.72	18.0	16.4
20(MQC)	− 1.47	− 8.50	− 3.31	− 4.52	3.84	4.65	− 18.3	17.0	− 18.9	9.69	19.6	18.7
60(HQC)	3.48	− 7.57	11.7	− 7.49	− 8.75	9.15	− 17.1	8.18	− 7.08	1.86	8.12	11.0

### Quantification of CLZ in the rat brain using MSI

**Fig. 4:**
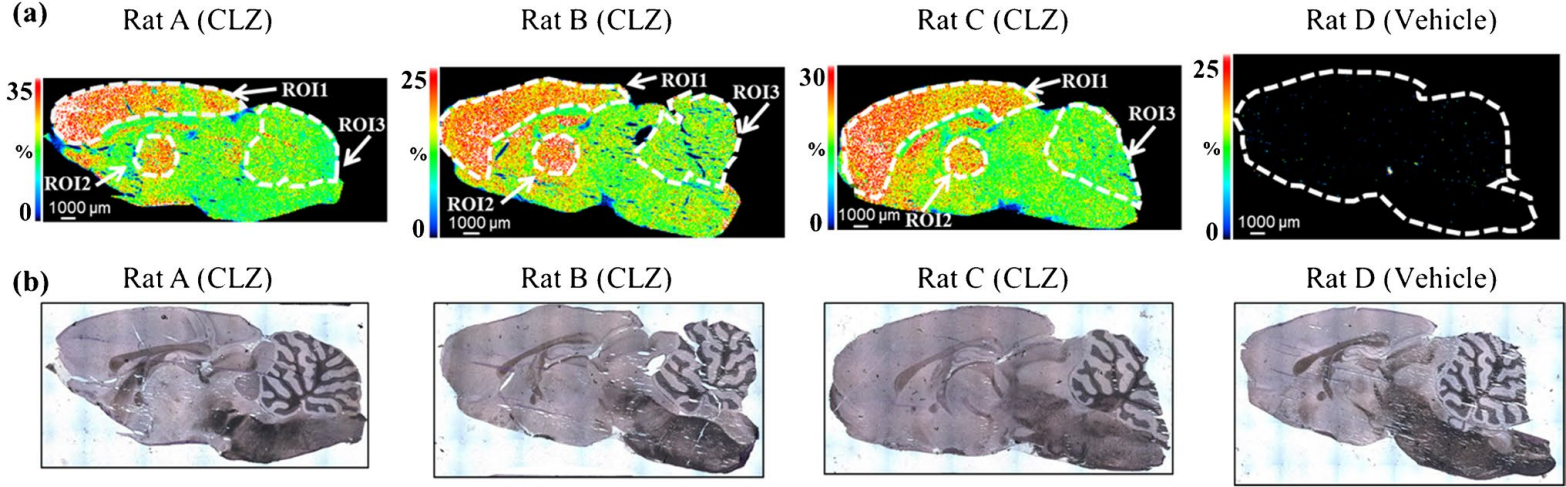
MSI image of clozapine (CLZ) and optical image of rat brain tissue sections after CLZ or vehicle treatment. Representative images were measured with a spatial resolution of 70 μm. The signals are normalized to the signal for the isotope-labeled standard (CLZ-d4). **a** Distribution of CLZ in the brain tissue sections from rats A, B, C, and D as determined using MSI at 70-μm resolution. **b** Optical images of the brain tissue sections from rats A, B, C, and D visualized using hematoxylin staining after MSI measurement. Corresponding selected regions of interest (ROIs 1, 2, and 3) show the ion distribution, localized to features of the regions defined using the Brain Atlas. The CLZ ion distribution was localized to a feature of the tissue

The application of QMSI to drugs was tested in three rat brains treated with CLZ and one brain treated with vehicle. The brain concentration of CLZ in rats is reported to reach maximum (approximately 40 μg/g) at 1 h after administration at 60 mg/kg [37]. Therefore, 1 h after administration as adequate exposure of the brain was chosen as the time of collection. Characteristic CLZ accumulation (Fig. 4a) was observed in each brain section obtained from rat brains A, B, and C. Three distinct regions, cortex, thalamus, and cerebellum, defined by hematoxylin staining and the Brain Atlas (https://mouse.brain-map.org/) were selected (Fig. 4a, b), and concentrations were calculated.

The calibration curves were assessed for linearity and accuracy in the concentration range of interest (1–80 μg/g), and were used to quantify the three ROIs in each rat brain section (Table S1). The accuracy of QC (*n* = 1) measured next to the calibration curve and the sample was 72.0% and 106% at 5, 20, and 60 µg/g, respectively (Table S1). The cortex and thalamus, the “hot spots” of drug accumulation in each individual, showed 1.40- to 1.73-fold higher concentrations relative to that in the cerebellum (Table 3).

**Table 3:** Quantification of clozapine (CLZ) in three regions of interest (ROIs) in the brain tissue from three rats using MSI

Animal ID	ROI	Region	MSI	Fold change
			Conc. (μg/g)	
A	1	Cortex	63.3	1.65
	2	Thalamus	54.2	1.42
	3	Cerebellum	38.3	1.00
B	1	Cortex	44.0	1.62
	2	Thalamus	47.0	1.73
	3	Cerebellum	27.2	1.00
C	1	Cortex	52.9	1.61
	2	Thalamus	46.2	1.40
	3	Cerebellum	32.9	1.00
Mean	1	Cortex	53.4	1.62*
	2	Thalamus	49.1	1.49*
	3	Cerebellum	32.8	1.00

Fold difference was calculated as ([ROI1 conc.] or [ROI2 conc.]) / [ROI3 Conc.]. The mean concentrations of ROIs were compared using the unpaired Student’s *t*-test (comparing cortex with cerebellum, and thalamus with cerebellum). The significance of differences was defined as *p < 0.05

The concentrations in the cortex in the present results are generally consistent with those measured by LC-Photodiode array detector in the brain (areas including the cortex and striatum) treated subcutaneously with 60 mg/kg CLZ [37]. They are also similar to MSI images of the distribution of CLZ in the brain, which are thought to reflect differences in the expression of the dopamine receptor D2 [38]. Although a single sagittal section does not provide a complete picture of the neural circuit, the accumulation of CLZ in the cortex and thalamus, important regions in the dopaminergic and glutamatergic neural circuits that are abnormal in schizophrenia, may provide insights into the yet unresolved pharmacological mechanism of action of CLZ [39, 40].

### Comparison of quantification between MSI and LCM-LC/MS

**Fig. 5:**
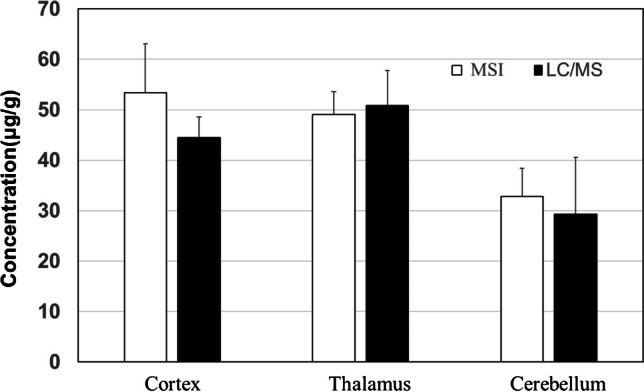
Comparison of clozapine (CLZ) quantification result in three regions of interest (cortex, thalamus, and cerebellum) in the brain tissue of rats (*N* = 3) using MSI (white) and LCM-LC/MS (black). The concentrations are shown as means ± SD

To validate the concentration of CLZ at each site obtained using MSI, the same sites as the selected ROIs, each concentration region of the calibration curve, and each concentration region of the QC were cut and recovered using LCM. After extraction, the concentrations were calculated using LC/MS (Fig. 5). The respective CLZ concentrations in the cortex, thalamus, and cerebellum measured using MSI and LC/MS were almost similar (Fig. 5). This suggests that the ability of MSI to directly and quantitatively measure CLZ in the brain is comparable to that of LC/MS, and that MSI is useful as a drug discovery tool to characterize the distribution of specific compounds that may lead to correctly understand drug efficacy and toxicity.

### Quantification of DA metabolites in the rat brain using MSI

**Fig. 6:**
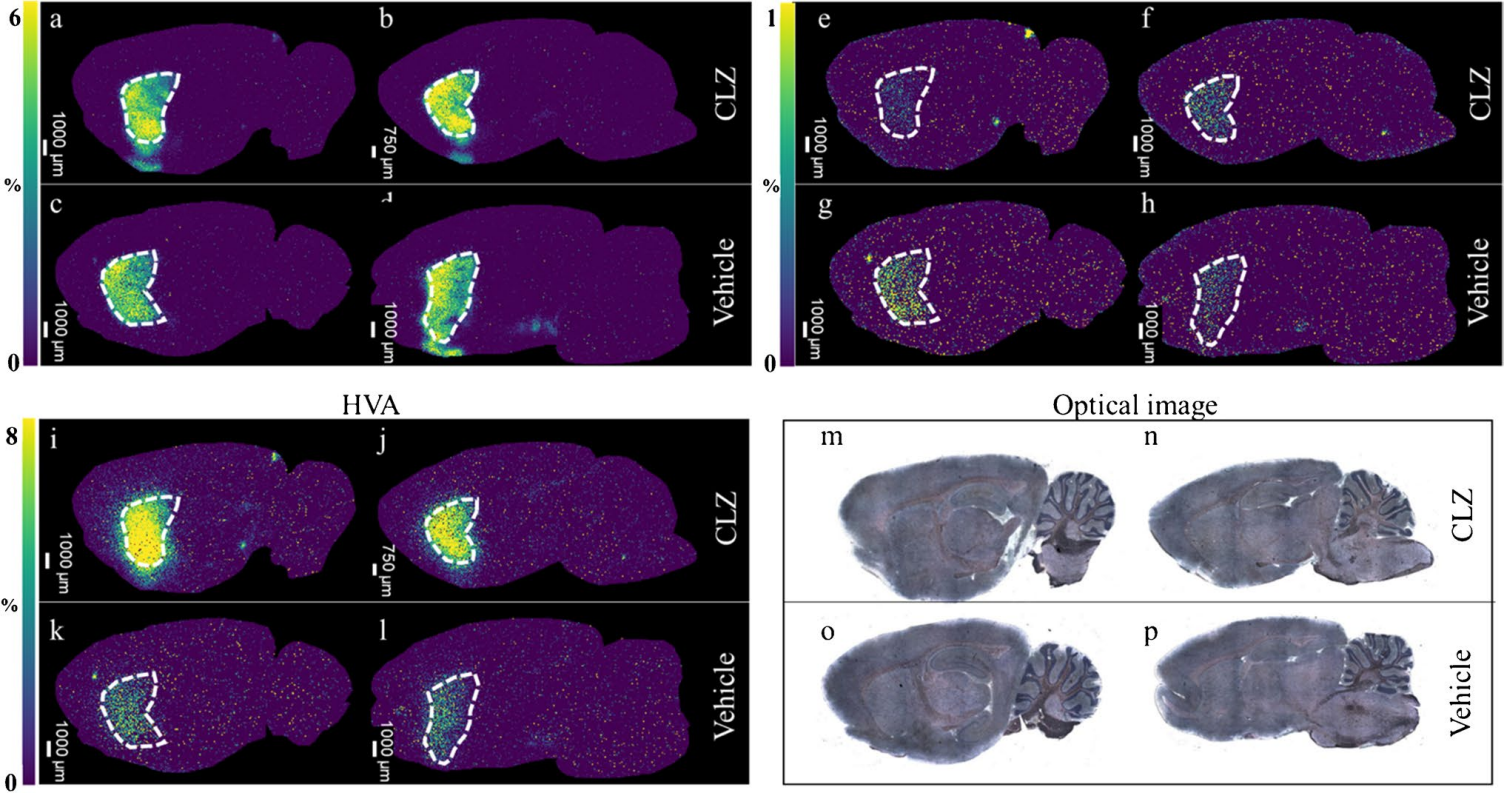
MSI images for dopamine (DA), 3-methoxytyramine (3-MT), and homovanillic acid (HVA) and optical images of rat brain tissue sections after treatment with clozapine (CLZ) or vehicle. Representative images were acquired with a spatial resolution of 70 μm. Images for DA, 3MT, and HVA (*m*/*z* 421.191, *m*/*z* 435.206, and *m*/*z* 450.170, scaled from 0 to 8%) were acquired and normalized to the signal for the isotope-labeled standard (*m*/*z* 425.217, *m*/*z* 439.232, and *m*/*z* 455.202). **a**–**d** Ion distribution of DA in the rat brain after administration of CLZ or vehicle. **e**, **f** Ion distribution of 3-MT in the rat brain after administration of CLZ or vehicle. **i**–**l** Ion distribution of HVA in the rat brain after administration of CLZ or vehicle. **m**–**p** Optical images of the rat brain tissue sections after administration of CLZ or vehicle visualized using hematoxylin staining after MSI measurement. Corresponding selected regions of interest (white dashed lines) show the ion distribution, localized to the striatum defined using the Brain Atlas

**Fig. 7:**
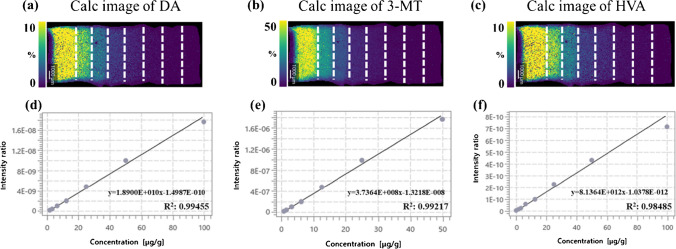
Calibration curves (Calc) of DA, 3-MT, and HVA with mimetic tissue models. **a** Calculated image of DA. **b** Calculated image of 3-MT. **c** Calculated image of HVA. **d** Calibration curves of DA. **e** Calibration curves of 3-MT. **f** Calibration curves of HVA. Images of DA, 3MT, and HVA (m/z 421.191, m/z 435.206, and m/z 450.170, scaled from 0 to 50%) were acquired and normalized to the signal for the isotope-labeled standard (m/z 425.217, m/z 439.232, and m/z 455.202). The region by white dashed lines indicates the respective concentration layer used for constructing the calc. weighted least squares calibration curves (solid lines) was applied to correlate the average ion intensity of each layer of the mimic model to its corresponding setting concentration

The derivatization method [14] was incorporated into the mimetic tissue model to validate the quantification of neurotransmitters. MSI images and concentrations obtained from each section of CLZ- and vehicle-treated brains are shown in Fig. 6 and Table 4. The calibration curve for DA showed good linearity in the concentration range of 1.56–100 μg/g with *R*^2^ > 0.995 and an accuracy calculated from QC samples (*n* = 2) with three concentrations measured after sample measurement was also good (< 15%) (Fig. 7a; Table S2).

**Table 4:** Quantification of dopamine (DA), 3-methoxytyramine (3-MT), and homovanillic acid (HVA) in the striatum of the rat brain after treatment with clozapine (CLZ) or vehicle using MSI

Molecules	Animal no	Clozapine	Vehicle
		Conc. (μg/g)	Conc. (μg/g)
DA	1	17.3	18.0
	2	18.7	20.7
3-MT	1	0.75**	0.47**
	2	0.55**	0.61**
HVA	1	1.51**	4.97
	2	1.30**	3.87

**Extrapolated value

The calibration curve for 3-MT showed a good linearity in the concentration range of 0.78–50 μg/g with *R*^2^ > 0.992 and an accuracy calculated from QC samples (*n* = 2) with three concentrations measured after sample measurement was also good (< 10%). The measured 3-MT concentration in the striatum was below the LLOQ, but the concentration was calculated by extrapolation (Fig. 7b; Table 4 and Table S2). The calibration curve for HVA showed good linearity in the concentration range of 1.56–100 μg/g with *R*^2^ > 0.985 and an accuracy calculated from the QC samples (*n* = 2) with three concentrations measured after the sample measurement was also good (< 20%) [41]. The HVA concentration in the striatum was slightly below the LLOQ in the vehicle group and, therefore, the concentration was calculated by extrapolation (Fig. 7c; Table 4 and Table S2). The concentration of DA and DA metabolites in the rat cerebellum used to create the calibration curve and QC samples was very low, and the ionic strength of the blank sample was about 1/15 to 1/10 of LLOQ. Thus, the effect on quantitation was considered to be extremely small. Although there is room for discussion regarding the calculation of concentration in the sample by extrapolation of concentration outside the calibration curve, it is considered acceptable. This is particularly true when considering that QMSI is often used in the drug discovery stage, where efficiency is primarily important for the rapid evaluation of drug distribution among a large number of compounds and the assessment of potential drug candidates. This issue of accurate calculation of concentration can be overcome by supplementing with other technologies such as LCM-LC/MS, as described in the previous discussion on CLZ.

Dopamine and its major metabolites are localized in the striatum, and when CLZ is administered to the rat, the concentration of HVA, the major metabolite of DA in the striatum, is greatly increased [42], whereas that of 3-MT is decreased [43]. We also found accumulation of DA, 3-MT, and HVA in the striatum (Fig. 6). The results (Table 4) show that DA concentrations in the striatum of rats ranged from 17.3 to 18.7 μg/g (vehicle group) and from 18.0 to 20.7 μg/g (CLZ group), 3-MT concentrations ranged from 0.55 to 0.75 μg/g (vehicle group) and from 0.47 to 0.61 μg/g (CLZ group), and HVA concentrations ranged from 1.30 to 1.51 μg/g (vehicle group) and from 3.87 to 4.97 μg/g (CLZ group). Previous studies have reported wide variability in the concentrations of DA and its metabolites in the rat striatum, with a concentration range of 0.0219 to 90.3 μg/g for DA, 0.0093 to 0.283 μg/g for 3-MT, and 0.0141 to 9.3 μg/g for HVA [44–48]. Direct comparisons are difficult because these studies differ in the rat strain used, age, and collection and measurement method (LC-electrochemical detection, LC-fluorescence detector, and LC/MS). However, even in these studies, the quantitative relationship among molecular species (DA > HVA > 3-MT) between the same measurements is consistent.

Tareke et al. performed LC/MS measurements on the rat striatum homogenates from the brain of Sprague–Dawley rats using stable isotopes [44] and reported a DA concentration of 18.0 μg/g, similar to that in this study. The concentrations of 3-MT (0.19 μg/g) and HVA (0.64 μg/g) in their study exhibited a 2- to threefold difference from this study, which could be due to the calculation of concentrations by extrapolation of the calibration curve in this study, or the difference in measurement of targets tissue condition, with LC/MS measuring the entire tissue while MSI measured a cut section [30]. In this study, the use of QMSI, which incorporates a derivatization reaction step, also allowed the measurement of the changes in concentration at a specific site, the striatum, for DA and its metabolites caused by compounds. Our results suggest that QMSI is a useful tool to explain site-specific changes in the concentration of biomarkers, such as neurotransmitters that may be associated with pathological changes.

There are a couple of notable challenges in applying QMSI to quantitative analysis of drugs and endogenous molecules in the brain. First, certain analytes are challenging to measure due to low concentration in samples or the lack of suitable MALDI reagents (such as optimal MALDI matrices for the analyte or analyte-specific derivative reagents for high sensitivity) for analytes. Secondly, in general, MSI has lower ion sensitivity compared to LC/MS. Additionally, simultaneous ionization without pretreatment in MALDI may sacrifice sensitivity, accuracy, and precision, raising concerns about quantitative reliability. Moreover, QMSI has lower throughput compared to LC/MS, influenced by factors such as the number of tissue sections on slide glass, space limitations of the slide glass, and measurement time based on spatial resolution settings. Understanding these limitations and characteristics is crucial in appropriately designing QMSI study protocols in drug discovery and development.

## Conclusion

We report on the optimization and performance evaluation of a reliable mimetic tissue model and its application to QMSI for drugs and endogenous metabolites. Our protocol significantly circumvents the difficulties encountered in creating a mimetic tissue block, simplifies the process, and saves space for calibration curve and QC samples. The protocol also enables quantitative analysis of drug in target tissues using MSI. We show that CLZ is predominantly localized in the cortex and thalamus rather than in the cerebellum. We also confirmed that the concentrations determined using QMSI are equivalent to those obtained from truncated target sites using LCM-LC/MS. QMSI can also be used for quantifying neurotransmitters employing derivatization methods. We elucidated the changes in concentration of DA and its metabolites in the striatum induced by CLZ. QMSI could be an indispensable method for reliable accurate data correctly interpreting drug effects and pathological conditions in drug discovery and development.

## Supplementary Information

Below is the link to the electronic supplementary material.

Supplementary file1 (DOCX 2046 KB)
