# Supplementary material for: Novel mimetic tissue standards for precise quantitative mass spectrometry imaging of drug and neurotransmitter concentrations in rat brain tissues
Source: Anal Bioanal Chem. 2024 Aug 10;416(26):5579–93. doi: 10.1007/s00216-024-05477-5 (PMC11493812; doi:10.1007/s00216-024-05477-5)
Supplement: Supplementary file 1 — Supplementary file1 (DOCX 2046 KB) [file 216_2024_5477_MOESM1_ESM.docx]

Novel Mimetic Tissue Standards for Precise Quantitative Mass Spectrometry Imaging of Drug and Neurotransmitter Concentrations in Rat Brain Tissues.

Kenichi Watanabe^1^, Sayo Takayama^1^, Toichiro Yamada^1^, Masayo Hashimoto^1^, Jun Tadano^2^, Tetsuya Nakagawa^2^, Takao Watanabe^3^, Eiichiro Fukusaki^4,5,6^, Izuru Miyawaki^1^, Shuichi Shimma^4,5,6*^

Supporting information


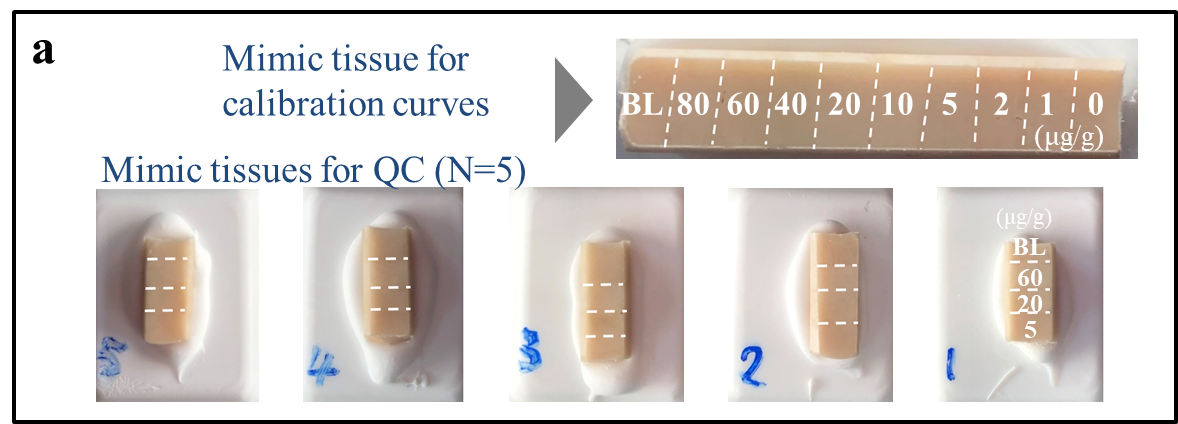


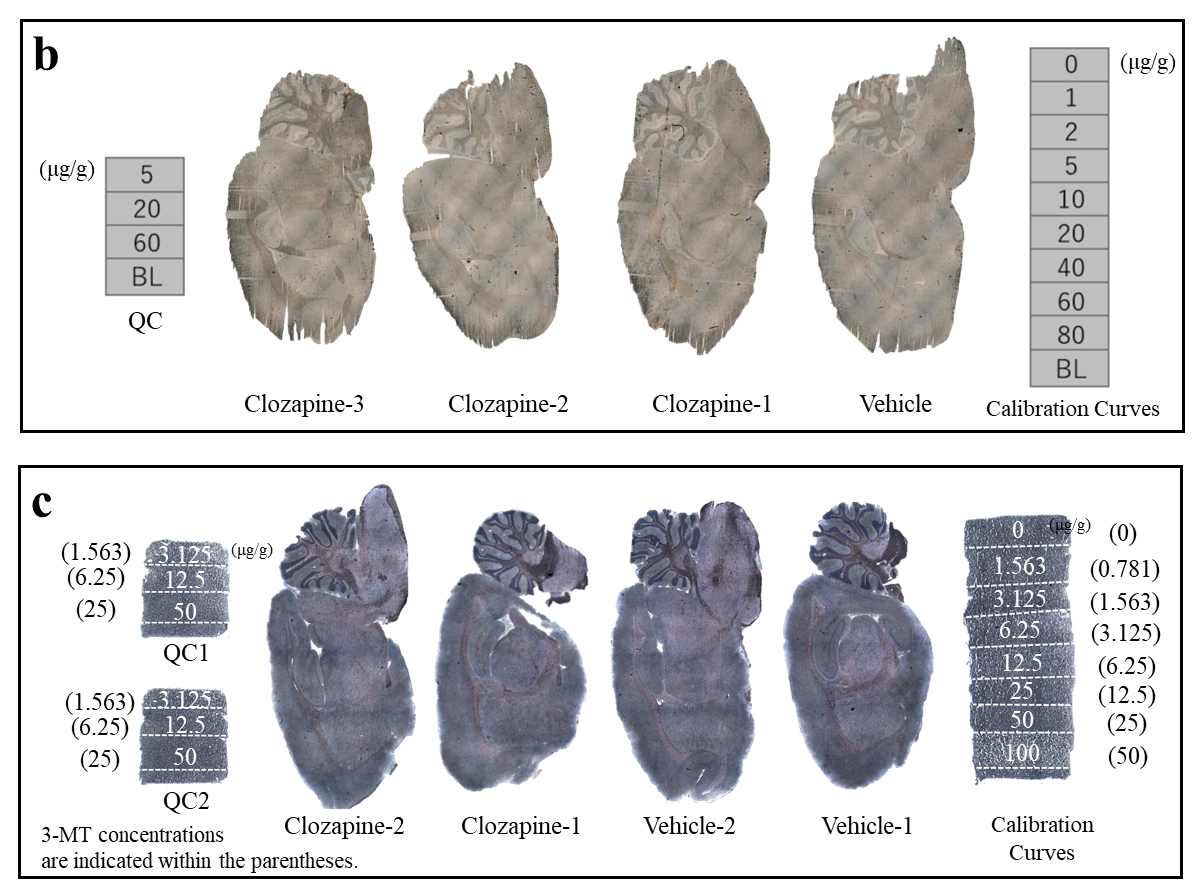


Figure S1. Position of sections on glass slide. a. Evaluation of mimetic tissue models. b. Quantification of clozapine. c. Quantification of the neurotransmitters.


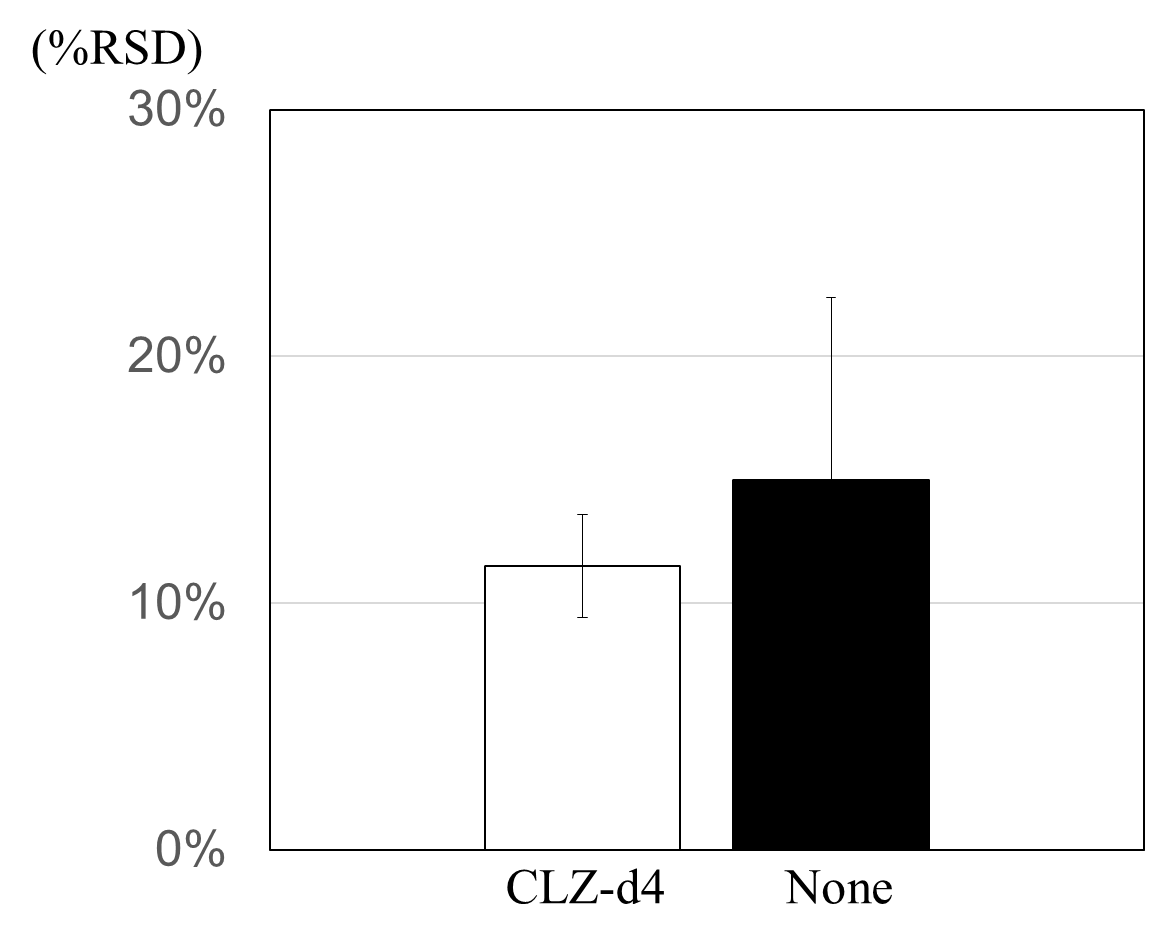


Figure S2. Effect of internal standard (CLZ-d4) on relative standard deviation (%RSD). The figure shows average %RSD for mimic tissues made from the brain sections across the three selected concentrations, using five replicates and two experiments with and without normalization by CLZ-d4.


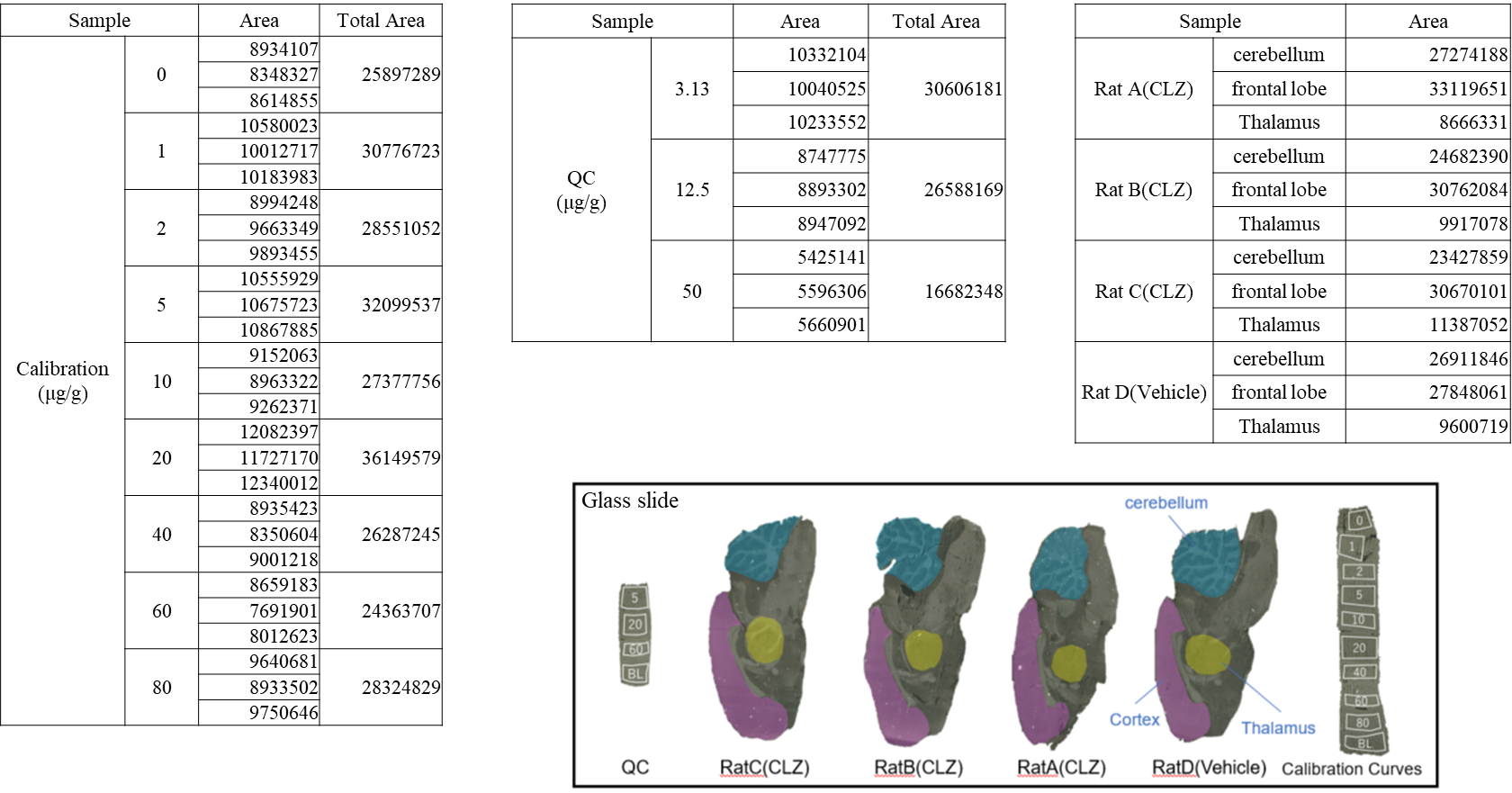


Figure S3: Area values of tissue sections from calibration curves, quality control samples (QCs), and laser-captured micro-dissected (LCM) rat brains.

During the quantification of the concentration of LCM-cut sections, the recorded area values (enclosed areas of Cortex, Thalamus, Cerebellum, Calibration curves, and QCs on the glass slides) were used to normalize the sample concentrations. To overcome the sensitivity limitation of LC/MS, three tissue sections of QCs and calibration curves were utilized, as the small area values could potentially impact the results. Clozapine lower limit of quantification (LLOQ) was 1 μg/g. It was selected with an analyte response which is at least 5 times the blank response.

Table S1. Linearity and accuracy of calibration curve and quality control (QC) for quantification of clozapine in three regions of interest in the brain tissue from three rats.

| Compound | Equation | R^2^ | Sample type | Concentration　(μg/g) | Accuracy (%) |
| --- | --- | --- | --- | --- | --- |
| Clozapine | y = 1.7356E+001x - 1.0651E+001 | 0.991 | Calibration Curve | 0.968 | -3.23 |
|  |  |  |  | 2.09 | 4.67 |
|  |  |  |  | 5.05 | 1.00 |
|  |  |  |  | 11.3 | 13.0 |
|  |  |  |  | 19.1 | -4.40 |
|  |  |  |  | 35.3 | -11.9 |
|  |  |  |  | 54.2 | -9.71 |
|  |  |  |  | 84.5 | 5.65 |
|  |  |  | QC | 4.09 | -18.2 |
|  |  |  |  | 17.5 | -12.4 |
|  |  |  |  | 43.2 | -28.0 |

Clozapine lower limit of quantification (LLOQ) was 1 μg/g. It was selected with an analyte response which is at least 5 times the blank response.

Table S2. Linearity and accuracy of calibration curve and quality control (QC1 and QC2) for quantification of neurotransmitters in the striatum of the rat brain tissue.

| Compound | DA | | 3-MT | | HVA | |
| --- | --- | --- | --- | --- | --- | --- |
| Equation | y = 1.8900E-004x - 1.4987E-004 | | y = 3.7364E-008x - 1.3218E-008 | | y = 8.1364E-012x + 1.0378E-012 | |
| *R^2^* | 0.995 | | 0.992 | | 0.985 | |
| Sample type | Concentration (μg/g) | Accuracy (%) | Concentration (μg/g) | Accuracy (%) | Concentration (μg/g) | Accuracy (%) |
| Calibration | 1.56 | 2.77 | 0.78 | 5.36 | 1.56 | -10.4 |
|  | 3.13 | -5.96 | 1.56 | -10.5 | 3.13 | -13.1 |
|  | 6.25 | 2.73 | 3.13 | 1.51 | 6.25 | 13.9 |
|  | 12.5 | -7.44 | 6.25 | -7.95 | 12.5 | -5.05 |
|  | 25 | 5.66 | 12.5 | 6.03 | 25 | 12.1 |
|  | 50 | 6.44 | 25 | 7.23 | 50 | 5.83 |
|  | 100 | -6.41 | 50 | -4.80 | 100 | -12.2 |
| QC1 | 3.13 | <0.1 | 1.56 | 4.47 | 3.13 | -17.5 |
|  | 12.5 | <0.1 | 6.25 | 0.982 | 12.5 | 1.90 |
|  | 50 | 0.121 | 25 | 7.90 | 50 | 7.93 |
| QC2 | 3.13 | <0.1 | 1.56 | 2.83 | 3.13 | -14.7 |
|  | 12.5 | <–0.1 | 6.25 | -5.32 | 12.5 | 0.515 |
|  | 50 | <0.1 | 25 | -3.92 | 50 | -1.93 |

DA, 3-MT, and HVA lower limit of quantification (LLOQ) was 1.56, 0.78, and 1.56 μg/g. It was selected with an analyte response which is at least 5 times the blank response.
